# Sensor-Based Precision Feeding Systems in Animal Production: Technologies and Applications

**DOI:** 10.3390/ani16091333

**Published:** 2026-04-27

**Authors:** Francesco Giannico, Claudia Carbonara, Anna Caputi Jambrenghi, Marco Ragni, Abdelfattah Zeidan Mohamed Salem, Simona Tarricone, Maria Selvaggi, Maria Antonietta Colonna

**Affiliations:** 1 Department of Human Sciences, Link Campus University, 00165 Roma, Italy; f.giannico@unilink.it; 2 Department of Medical and Surgical Sciences, University of Foggia, 71122 Foggia, Italy; claudia.carbonara@unifg.it; 3 Department of Soil, Plant and Food Sciences, University of Bari Aldo Moro, 70126 Bari, Italy; anna.caputijambrenghi@uniba.it (A.C.J.); marco.ragni@uniba.it (M.R.); abdelfattah.salem@uniba.it (A.Z.M.S.); maria.selvaggi@uniba.it (M.S.); mariaantonietta.colonna@uniba.it (M.A.C.)

**Keywords:** PFS—precision feeding system, animal production, ruminants, monogastric, poultry, aquaculture, ICT—information and communication technologies

## Abstract

Despite the productivity and economic limitations imposed by environmental and climatic conditions, livestock systems play a fundamental role in preserving habitats and high-conservation-value species, while delivering a broad spectrum of ecosystem services to rural populations. Breeders need timely information to produce safe, inexpensive, environmentally, and welfare-friendly food products. Information on feeding and nutrition is of particular importance since it represents a significant percentage of animal breeding costs. Automating the collection, analysis, and use of production-related information on livestock feeding systems represents one of the central challenges facing the sector. Precision feeding systems (PFSs) have deeply changed farm management by providing new information on the health status of animals, their welfare, and nutritional requirements. PFSs encompass modern electronic and ICT-related (information and communication technologies) technologies that facilitate the electronic measurement of critical components, ensuring optimum efficiency of both resource use and animal productivity. This review analyzes the current state and potential applications of precision feeding systems for sustainable livestock production. The implementation and feasibility of PFSs have been investigated across the major animal production species and contexts. Based on the available literature, real-time monitoring and control systems can improve the production efficiency of livestock farms. However, further research is needed, as several components of PFSs are still at different stages of development and commercial readiness.

## Simple Summary

Livestock farming is important for local economies and rural communities, providing valuable ecosystem services and supporting habitat conservation, despite challenges due to harsh environmental and climatic conditions. One of the main costs in animal production is feeding and nutrition. To manage this more efficiently, precision feeding systems (PFSs) use modern electronic and digital technologies to monitor animal health and nutritional needs in real time, helping farmers make better decisions and improve overall farm performance. PFS tools offer great potential in several animal species and livestock systems. They can boost productivity, reduce labor, and promote more sustainable farming practices. However, adoption of these technologies is still limited. Many PFS tools are not yet fully developed, and clear strategies for bringing them to market—in collaboration with the private sector—are still lacking. More research is needed to make these innovations practically available to farmers working in the most difficult and resource-limited environments, ensuring that farming systems can benefit from technological progress.

## 1. Introduction

The continuous expansion of the global population is placing unprecedented pressure on food production systems worldwide. Over the next five decades, consumption of animal-derived products is projected to increase by 17%, while the global demand for animal protein is expected to double by 2050 [1,2,3,4]. Meeting these demands in a sustainable manner will require a fundamental shift toward production systems that maximize efficiency while minimizing environmental impact.

Central to this challenge is the need to align animal nutrient requirements precisely with the nutritional composition of feed. Animal welfare and productive performance are inherently dependent on adequate and consistent nutrient supply [5]; however, achieving this in practice is complicated by two principal sources of variability: differences among individual animals within a herd or flock and fluctuations in the nutrient concentration of feedstuffs within a given batch [6,7]. These variations in total mixed ration (TMR) composition can exert significant effects on animal health and productivity, with downstream consequences for farm profitability and environmental sustainability [8]. Critically, while the manifestation of these challenges differs across species and production contexts, their underlying nature is shared across polygastric species—such as dairy and beef cattle, sheep, and goats—as well as monogastric species including swine and poultry, and extends equally to aquaculture systems.

Precision feeding systems (PFSs) have emerged as a promising technological response to these shared challenges. By integrating innovative sensors and data-driven tools, PFSs enable the fine-tuned matching of nutritional supply to individual animal requirements across a wide range of species, thereby improving both resource utilization and animal welfare. PFSs are widely regarded as a core component of the broader precision livestock farming (PLF) framework, which encompasses all technologies designed for continuous, automated, real-time monitoring of individual animals and their production environment [9]. Through the measurement of physiological, behavioral, and production-related indicators, PFSs support enhanced management strategies and improved overall farm performance [10], whether applied to a dairy herd, a swine unit, a poultry house, or a fish farm. In the context of modern, highly industrialized animal production—where individual stockpersons oversee large numbers of animals—such systems play a critical role in enabling timely detection of health issues and welfare concerns that would otherwise go unnoticed through manual observation alone [11]. Moreover, by reducing waste and optimizing the number of animals needed to achieve a given level of output, PFSs contribute meaningfully to both economic and environmental sustainability across all production systems [12]. The capabilities of PFSs are further expanded through integration with complementary technologies, including near-infrared (NIR) spectroscopy, machine learning algorithms, and Internet of Things (IoT) platforms [13]. Although much of the foundational research in this field has been conducted in dairy cattle—where, for instance, Piccioli-Cappelli [14] demonstrated that NIR scanner-based PFS significantly improved TMR compositional consistency, resulting in better nutrient utilization and reduced production costs—the principles and technologies underpinning these systems are broadly transferable. Indeed, the rapid advancement of sensor technologies, artificial intelligence, and digital connectivity is progressively broadening the applicability of PFSs to species and production contexts that have, until recently, received comparatively little systematic attention.

**Figure 1:**
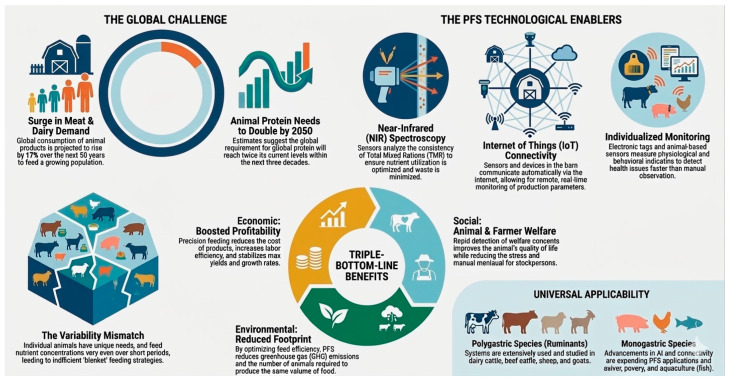
PFSs in animal production.

The present review therefore aims to bridge this gap by providing a comprehensive and critical synthesis of the current state of sensor-based precision feeding systems across the full breadth of animal production. This encompasses both polygastric species—dairy cattle, beef cattle, sheep, and goats—and monogastric species—swine and poultry—as well as farmed fish, reflecting the recognition that sustainable intensification of animal production is a challenge that cuts across taxonomic and production boundaries. Particular attention is given to available technologies, species-specific applications, current limitations, and future perspectives for the development of more efficient, sustainable, and welfare-oriented production systems (Figure 1).

## 2. Materials and Methods

### 2.1. Literature Search Strategy

A structured review of the literature was carried out to evaluate the state of the art of the application of precision feeding systems (PFSs). The research was conducted on Web of Science^®^, Scopus^®^, and PubMed^®^, focusing on studies carried out in the last fifteen years (from 1 January 2010 to 15 February 2026). This bibliographical review was carried out to identify innovative PFSs in the farming of monogastric and polygastric animals and in aquaculture. The keywords and search strings used for the literature research were combined using Boolean operators (AND, OR, NOT), and finally they were organized into four macro-thematic clusters, as reported in Table 1.

### 2.2. Eligibility Criteria

Studies were selected according to the Population, Intervention, Comparison, Outcome, Study (PICOS) design framework, as reported in Table 2. Regarding population, studies involving monogastric (swine, poultry and laying hens), polygastric animals (dairy cattle, beef cattle, sheep, goats) and aquaculture were eligible. Concerning intervention, only studies implementing sensor-based or automated precision feeding technologies were considered eligible. Studies focused exclusively on dietary formulation without any technological component were excluded. As for comparison, studies comparing PFSs with conventional feeding approaches or evaluating different PFS technologies were prioritized. The outcomes of interest included feed intake, feed efficiency, feeding behavior, nutrient utilization, body condition score, animal welfare, and economic performance. With respect to study design, peer-reviewed original research articles, systematic reviews, meta-analyses, and book chapters published in English between 2010 and 15th February 2026 were included. Given the heterogeneity of study designs, methodologies, and reported outcomes across the retrieved literature, a quantitative meta-analysis was not deemed appropriate. Instead, a narrative synthesis approach was adopted, through which studies were grouped thematically to identify recurring patterns and key drivers of PFSs in polygastric and monogastric animals and in aquaculture technologies applied in PFS. Particular emphasis was placed on the interactions among these factors and their implications for different species breeding systems. The synthesis was conducted within a holistic framework, acknowledging the inextricable interconnection between animal health, feeding system, animal production and environmental integrity.

**Figure 2:**
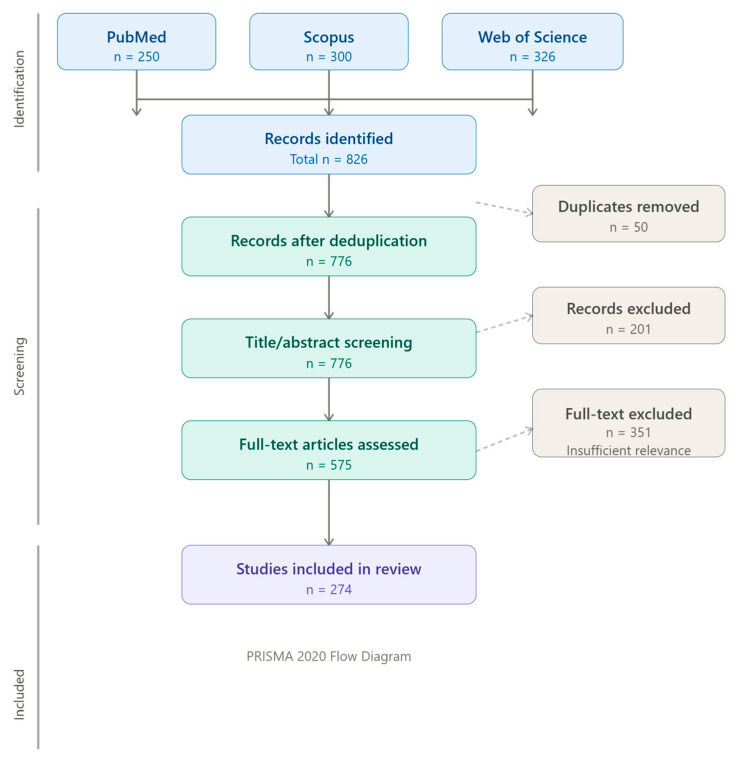
PRISMA 2020 flowchart diagram.

Conference abstracts, proceedings without peer review, studies without a retrievable full text, and opinion pieces were excluded. After the removal of 50 duplicates through the Zotero program, titles and abstracts were screened for relevance, followed by full-text reading of selected articles. The thematic classification of retrieved studies was performed independently by three authors, and any discrepancies in categorization were resolved through discussion and consensus among all co-authors. A total of 826 records were initially retrieved. After screening, 201 articles were excluded based on title and abstract, and 351 were further excluded after full-text reading due to insufficient relevance or methodological detail. Ultimately, 274 studies were included and discussed in the present review (Figure 2).

## 3. Definition and Classification of Precision Feeding Systems

Precision feeding systems (PFSs) can be defined as animal-tailored integrated technological approaches designed to deliver the correct amount of feed, characterized by the needed nutritional composition, at the right time [15]. Within precision livestock farming (PLF), PFSs specifically address the nutritional dimension, linking individual animal data to feeding decisions through automated control systems [16]. Unlike conventional feeding strategies, which typically provide uniform rations to groups of animals based on average requirements, PFSs operate at the individual animal level, accounting for intra- and inter-individual variability in nutrient requirements, feed intake behavior, physiological status, and productive performance. The shift from group-based to individual-based feeding represents a paradigmatic change in animal nutrition management, enabled by the convergence of sensor technologies, data analytics, and automation [16].

### 3.1. Classification

PFSs can be classified according to different criteria, including the type of technology employed, the level of automation, and the target species. Based on these dimensions, four main categories can be identified. To provide a structured framework, the present classification organizes PFS components into four categories based on their primary functional role within the feeding management pipeline: (i) data acquisition, (ii) physical feed delivery, (iii) data processing and decision making, and (iv) system integration and connectivity. While individual technologies may contribute to more than one function, each category is defined by its predominant role, and the boundaries between categories reflect logical stages in the PFS workflow rather than mutually exclusive technological domains.

#### 3.1.1. Sensor-Based Monitoring Systems

These systems’ purpose is data acquisition and collecting real-time information on animal status and behavior. They rely on a variety of sensors to continuously collect data on individual animals, and these data are used to inform feeding decisions. Key sensor types include the following:

- Radio-frequency identification (RFID) tags for individual animal identification at feeding stations [17].
- Accelerometers and gyroscopes worn as ear tags, leg bands, or collars, used to monitor feeding behavior, rumination activity, and physical activity patterns [5,17].
- Rumen boluses—electronic devices ingested by ruminants that measure rumen pH, temperature, and motility in real time [18].
- Load cells and feed intake sensors integrated into feeders to measure individual feed consumption with high precision [19].
- Near-infrared (NIR) spectroscopy sensors for real-time analysis of feed composition, including dry matter, protein, and fiber content [14].

Although load cells and NIR sensors are physically embedded in feeding equipment, they are classified here because their predominant function is data collection rather than feed delivery.

#### 3.1.2. Automated Feeding and Delivery Systems

The primary function of these systems is to deliver feed physically and to translate nutritional recommendations into precise mechanical actions:

- Automatic milking systems (AMSs) with integrated concentrate dispensers allow individualized concentrate allocation based on milk yield and stage of lactation in dairy cattle [20].
- Electronic sow feeding (ESF) stations for group-housed sows enable individual feed allocation based on body condition, parity, and reproductive stage [21].
- Robotic total mixed ration (TMR) systems are capable of preparing and delivering customized rations with variable ingredient proportions based on real-time NIR analysis [13].
- Demand feeders and self-feeders equipped with sensors to detect feeding initiation behavior are used extensively in aquaculture and swine production [22,23].

It should be noted that several of these systems (e.g., AMS, ESF stations) also incorporate sensing and decision-support functions; however, their defining characteristic within PFSs is the physical, individualized delivery of feed.

#### 3.1.3. Decision Support and Modeling Systems

These systems process the data collected by sensors and feeding equipment through mathematical models and algorithms to convert raw data into individualized feeding recommendations:

- Mechanistic nutritional models simulate nutrient digestion, absorption, and utilization based on animal-specific parameters [7].
- Machine learning (ML) and artificial intelligence (AI) algorithms are capable of predicting individual feed intake, body weight gain, or milk yield from multi-sensor data streams [24,25].
- Farm management information systems (FMISs) integrate feeding data with health, reproductive, and production records to support holistic decision making [26,27].

Unlike the systems in the previous categories, the components described here operate primarily at the software and analytical layers, with no direct role in data acquisition or feed delivery.

#### 3.1.4. Connectivity and Integration Platforms

The full potential of PFSs is realized when individual components are interconnected through digital infrastructure. This category differs from the preceding three in that it does not perform a specific feeding-related function autonomously but rather enables the other components to operate as a coherent, integrated system:

- Internet of Things (IoT) platforms enable real-time data transmission from barn-level sensors to cloud-based management systems [28].
- Edge computing solutions allow data processing to occur locally, reducing latency and dependency on stable Internet connectivity—particularly relevant in remote or semi-intensive production environments [29].
- Interoperability standards and APIs facilitate data exchange between different PFS components and third-party software platforms [30].

## 4. Precision Feeding Systems in Animal Production

Understanding animal behavior is essential to effectively managing their nutrition and movements [31]. Several researchers have conducted extensive studies on flavor preference, which can be used in dietary formulations [8,32,33]. It is commonly believed that ruminant livestock lack the ability to form long-lasting flavor aversions. However, research has shown that ruminants possess a gut–brain system that can form distinct and long-lasting flavor dislike, similar to monogastric animals [34]. An aversion is defined as a decrease in preference for food after consuming it because of the taste, odor, texture, and post-ingestive effects unique to each food [35]. The mechanism for flavor aversion is initiated by the neuronal connections between the gut and the brain’s emetic center, which operates independently of the animal’s consciousness or cognitive abilities [36]. Animals tend to avoid foods that induce gastrointestinal pain when they are exposed to the same flavor later on. This is because exposure to the same flavor stimulates the emetic system [37]. The flavor aversion mechanism evolved as a result of animal interactions with hazardous plants and has adaptive benefits [38]. However, it is not a perfect method, especially when natural correlations between flavor and gastrointestinal repercussions are modified to suit human aims other than animal nutrition optimization, such as crop and tree protection. Even if a crop is edible, flavor aversion can occur if livestock is injected with a chemical that causes gastrointestinal discomfort after they sample new crops as novel foods [39,40].

Operant conditioning, also known as instrumental learning, is the repetition of effective behavior when animals are repeatedly exposed to the same setting and the eradication of ineffective behaviors [41]. Domestic animals can learn to perform extremely complex behaviors through operant conditioning, and they can develop high levels and intensity of responses [42]. Cattle and sheep have been trained to perform difficult spatial and foraging tasks using positive and negative reinforcement [43]. Studies conducted by Hirata et al. [44] and Ksiksi and Laca [45] showed that food rewards were strongly linked to visual signals such as clover sods and colored flags in sheep and cattle. Cattle demonstrated the ability to respond to the spatiotemporal character of food rewards with various foraging tactics [46], while sheep used spatial memory to perform localized searches when food was predictable in time and place but searched systematically when food was unpredictable [47]. When sheep learned the locations of food through experience, they first sought in familiar locations and then employed visual cues [48]. In addition, Langbein et al. [49] found that goats can learn operant discrimination tasks when they have self-regulated access to an automated device. Goats improved their ability to learn new symbols when they were subjected to various activities, which suggests that they acquired “learning sets” or the ability to learn. Furthermore, Baymann et al. [50] found that animals in stable groups and social surroundings learned best.

Beyond the general mechanisms of learning and conditioning, substantial individual variability in behavioral traits—including personality, temperament, and coping style—has been documented across livestock species, and this variability has direct implications for the practical implementation of precision livestock technologies. In the context of automated milking systems (AMSs), individual differences in personality traits such as boldness, activeness, and fearfulness have been shown to significantly influence both the rate and quality of adaptation. Cows exhibiting greater fearfulness toward novel stimuli were less likely to voluntarily access the full concentrate allowance available in the AMS, resulting in reduced total dry matter intake and greater day-to-day variability in feed consumption [51]. Conversely, cows scoring high for boldness and activeness adapted more readily to the AMS immediately after calving, displaying higher milk yield and more frequent voluntary milking visits during the early post-introduction period [52]. Similarly, temperament assessed prior to system changeover was associated with productive performance during the transition, with reactive cows outperforming calmer individuals in milk yield during the first days in the AMS [53]. The influence of personality on adaptation has also been observed during the training phase: more active cows showed a markedly higher risk of kicking off teat cups during AMS training, though this disadvantage diminished substantially over the weeks following training completion [54]. Collectively, these findings indicate that individual behavioral traits are not merely sources of noise in production data but constitute meaningful biological variables that modulate how animals engage with, learn from, and benefit from automated feeding and milking technologies [55]. Accounting for such inter-individual variability is therefore an important consideration in the design and management of precision feeding systems, particularly when voluntary animal interaction with automated equipment is central to system performance.

Precision feeding systems represent a technological evolution in the nutritional management of livestock, allowing the optimal use of feed through continuous and personalized monitoring of the specific needs of each animal. Precision nutrition allows for feeding animals with personalized diets on a daily basis, utilizing feeding techniques that enable the provision of the right amount of feed with adequate composition at the appropriate time to individual animals or groups of animals [8]. The integration of mathematic models and artificial intelligence facilitates the analysis of wide datasets, considering factors such as animal physiology, genetics, and environmental conditions, allowing for the determination of intricate patterns within these datasets and leading to a unique level of precision in feed formulation [56]. Implementation of precision nutrition represents a paradigm shift toward sustainable livestock farming, offering significant potential to optimize production efficiency, reduce costs, and minimize environmental impact through reduced nutrient waste [57]. The effective design of precision feeding systems does not rest on technology alone but emerges from the convergence of behavioral science and engineering. The behavioral and cognitive mechanisms described in the preceding sections—including operant conditioning, spatial memory, social learning, flavor preference, and individual personality traits—are not merely theoretical constructs: they constitute the biological substrate upon which automated feeding systems must operate. Understanding how animals learn to interact with novel equipment, how they respond to feed-associated stimuli, and how inter-individual differences in temperament modulate voluntary system use is prerequisite knowledge for translating sensor outputs and algorithmic recommendations into effective nutritional interventions. Conversely, sensing technologies amplify the practical reach of behavioral science by providing continuous, objective, and individual-level data streams that would be impossible to obtain through direct observation alone. RFID-based identification, accelerometers, and intake sensors can detect subtle deviations in feeding behavior—such as reduced visit frequency, shortened meal duration, or altered rumination patterns—that reflect underlying physiological or psychological states, enabling timely and targeted responses. In this integrated framework, behavioral knowledge informs system design (e.g., the placement of feeding stations, the timing and magnitude of concentrate rewards, the pacing of training protocols), while sensing data continuously refine the behavioral picture of each individual animal, creating a feedback loop between biological reality and technological response.

This approach is facilitated by advanced technologies such as sensors and automation systems that monitor feed intake and animal growth in real time, and by optimizing feed efficiency, precision feeding reduces feed waste and nutrient excretion, which in turn reduces the environmental footprint of livestock production [58]. Available technologies include automated systems for individual feed intake quantification, feeding behavior monitoring, rumination pattern tracking, and pasture biomass calculation [59]. Wearable and biometric sensors—such as smart ear tags, rumination collars, and boluses—continuously measure activity, temperature, and feeding patterns, enabling early detection of lameness, fever, or reduced intake far before clinical symptoms appear [60]. Advanced systems utilize artificial intelligence to predict livestock feed intake and provide feeding recommendations, generating daily quantitative feeding predictions based on hundreds of data points collected from computer vision systems and external data sources, accounting for feeding rates, feeding times, feeding cycles, livestock behavior, ration type, weather, and other factors [61,62].

This scientific approach to precision feeding complements the behavioral and cognitive mechanisms described in the preceding sections, demonstrating how technological advances can leverage animals’ natural learning abilities and behavioral patterns to optimize nutritional management while addressing contemporary challenges in sustainable livestock production.

### 4.1. Polygastric Animals

#### 4.1.1. Dairy Cattle

The livestock industry is slowly adopting new technologies despite their widespread availability [11,63].

In dairy farming, diet formulation is typically based on dry matter (DM) content; however, in practice, feeds are added to the total mixed ration (TMR) based on their as-fed weight. This discrepancy introduces significant variability in the nutritional composition of the delivered ration. Surveys conducted on high-yielding dairy herds have documented that forage inclusion in TMR diets ranged from 45 to 53% on a DM basis, with corn silage representing 41–68% of the DM fraction [64,65,66]. Alfalfa and corn silage samples collected within commercial farms showed marked variability in DM concentration over both short- and long-term periods [6]. The varying moisture content of wet feeds, particularly silages, affects the amount of DM delivered and its nutrient composition, which in turn influences ingredient palatability and sorting behavior [39,67]. This generates additional variability in dry matter and nutrient intake, leading to individual nutrient imbalances and altered fermentable energy intake, which has been shown to negatively affect milk cheese-making properties [68,69]. These effects are particularly relevant in specialized intensive systems, such as farms producing milk for Protected Designation of Origin (PDO) cheeses, where corn silage represents the primary forage base with average inclusion rates of approximately 23 kg/head per day for lactating cows [70,71]. To address TMR variability, several sensor-based PFS solutions have been developed and validated in intensive dairy systems. Among these, a system based on a near-infrared (NIR) scanner mounted on the scraper of a front miller has been proposed to perform real-time DM analysis of each ingredient, allowing automatic adjustment of ingredient loads from as-fed to DM weight [14]. This approach improves TMR consistency, maintaining a more constant nutritional composition of the distributed ration, which contributes to the reduction in metabolic disorders—particularly critical during the early lactation period—and to better efficiency of nutrient utilization and reduced production costs [14]. Table 3 shows some of the most applied technologies in dairy cattle, along with the parameters monitored.

#### 4.1.2. Beef Cattle

Conventional feeding strategies in beef cattle production rely on group-based ration formulation, which inherently fails to account for inter-individual variation in nutrient requirements, metabolic efficiency, and growth trajectory [86]. The advent of precision livestock farming (PLF) has catalyzed the development of precision feeding systems capable of delivering individualized dietary inputs, thereby maximizing feed conversion efficiency while minimizing environmental externalities [87,88]. Precision feeding is broadly defined as the real-time adjustment of nutrient supply to match the dynamic physiological requirements of individual animals, reducing the gap between nutrient intake and actual metabolic demand [89]. In the context of beef cattle, this paradigm shift holds particular relevance given the substantial contribution of enteric fermentation and nitrogen excretion to greenhouse gas emissions and environmental nitrogen loading. Costa et al. [90] highlighted the potential of sensor technologies to monitor lying behavior, step activity, and rumination in calves, parameters that can be useful in detecting behavioral changes indicative of disease, response to painful procedures, or positive welfare states such as play behavior.

The nutritional requirements of beef cattle are not static [91]; they fluctuate as a function of body weight, growth phase, health status, breed, sex, and environmental conditions [92]. Traditional total mixed ration (TMR) systems deliver uniform nutrient concentrations to entire pens, inevitably resulting in both underfeeding and overfeeding within the same cohort. Individual variation in dry matter intake (DMI), residual feed intake (RFI), and average daily gain (ADG) has been extensively documented in the literature [93,94,95]. Animals with low RFI—a heritable trait indicative of superior feed efficiency—consume significantly less feed than cohorts of equivalent performance, underscoring the biological heterogeneity that group feeding strategies cannot address [96]. Precision feeding systems exploit this variability by calibrating ration composition and delivery volume at the individual level, with the aim of reducing feed waste and improving nitrogen use efficiency (NUE) [97]; however, the degree to which true individual-level adjustment is achievable in routine commercial practice remains variable and context-dependent, and many currently deployed systems operate at sub-group rather than strictly individual resolution.

The operational backbone of any PFS is a robust animal identification system [98]. Radio-frequency identification (RFID) ear tags and electronic boluses provide continuous, non-invasive individual recognition as animals approach feeding stations and have been validated in both research and commercial settings [17,99,100]. These systems interface with automated feed dispensers capable of modifying ration composition based on pre-programmed nutritional algorithms, a functionality that is well established in research platforms but whose implementation in large-scale commercial feedlots remains limited by infrastructure costs and management complexity [17]. Complementary sensing technologies—including accelerometers for behavioral monitoring, rumination sensors, and infrared thermography for early disease detection—generate additional data streams that can inform ration adjustments, though their integration into fully automated, real-time decision pipelines is more commonly reported in experimental than in routine farm settings. Individual automated feeding stations—commercially exemplified by systems such as RIC (Roughage Intake Control) and Insentec feed bins—record with high precision the identity of the feeding animal, visit duration, feed disappearance, and meal frequency [101,102] and represent among the most mature and widely validated tools for individual-level intake monitoring currently available. Integration with weigh platforms allows concurrent body weight monitoring, enabling continuous updates to feeding models without manual intervention, although such integrated configurations are predominantly reported in research facilities. Central to PFS functionality is a decision support system (DSS) that integrates phenotypic data, growth models, and nutritional databases to generate individualized feeding recommendations [103,104]. Machine learning algorithms trained on large datasets of cattle performance metrics have demonstrated superior predictive accuracy compared to static factorial models, particularly in dynamic feedlot environments [105,106], though their transferability to diverse commercial contexts requires further validation.

The potential environmental co-benefits of precision feeding in beef systems have received growing attention, though the evidence base warrants careful interpretation [107]. Studies conducted primarily under research conditions suggest that more accurate matching of dietary crude protein to individual requirements may reduce urinary nitrogen excretion—the principal precursor of ammonia volatilization and nitrous oxide emissions from manure—with reductions in the range of 15–25% reported under controlled experimental protocols [108,109]. It should be noted, however, that these estimates derive largely from nutritional optimization trials rather than from precision feeding implementations per se, and their translation to commercial-scale outcomes has not yet been systematically demonstrated. Similarly, there is experimental evidence suggesting that optimizing the energy-to-fermentable carbohydrate ratio on a per-animal basis may contribute to reducing enteric methane emissions per unit of beef produced [110,111], although the magnitude and consistency of this effect under field conditions remain to be established. Realizing these environmental benefits at scale will require not only the technical refinement of precision feeding platforms but also robust on-farm validation studies that can confirm whether laboratory and research-station findings can hold under the operational constraints of commercial beef production. Table 4 shows some of the most applied technologies in beef cattle, along with the parameters monitored.

#### 4.1.3. Small Ruminants

The identification and classification of feeding behavior in ruminant species represents a fundamental prerequisite for improving the efficiency of animal production [124,125]. Direct behavioral observation, while accurate, requires extensive labor, is time-consuming, and is often not feasible under commercial farming conditions [126,127]. Early measurement approaches, such as the Vibracorder device used to record sheep head movements during grazing [128], have been progressively superseded by digital sensor technologies, driven by advances in cloud computing, artificial intelligence, and miniaturized electronics [129,130]. Among currently available technologies, tri-axial accelerometers have been extensively validated for the automated classification of sheep behavior [131,132]. Alvarenga et al. [133] assessed the accuracy, sensitivity, specificity, and precision of five tri-axial accelerometer-derived variables—mean *X*-axis acceleration, inclination, pitch, movement variation, and *Z*-axis acceleration—for the identification of sheep behavior at pasture. Their results demonstrated that accelerometers placed under the jaw of sheep could reliably discriminate grazing from non-grazing activity, with the natural log-transformed mean *X*-axis acceleration providing the most robust classification across different epoch lengths. It is important to note, however, that accelerometers and similar wearable sensors function primarily as behavioral monitoring tools: they generate continuous descriptive data on activity and feeding patterns but do not in themselves enable individualized ration adjustment or nutrient delivery. Their value within a precision feeding workflow lies in their capacity to serve as inputs to decision support systems, flagging anomalies in feeding behavior—such as reduced grazing time or altered rumination patterns—that may trigger a nutritional or health management response.

Radio-frequency identification (RFID) technology occupies a distinct and more directly operational role within precision feeding systems for small ruminants. Unlike accelerometers, RFID-based identification constitutes the enabling layer of individualized feed delivery: by recognizing the specific animal accessing a feeding station, RFID systems allow automated dispensers to retrieve that animal’s nutritional profile and adjust ration composition or quantity accordingly in real time [134]. Vaintrub et al. [13] highlighted the capacity of RFID systems to store large datasets for various decision-making processes, including individualized production and feeding management. Passive electronic identification (EID) tags, which integrate electronic weighing, recording, and drafting equipment based on a simple information code and a copper coil, are particularly well suited for sheep farming due to their small size and the absence of battery requirements [135]. Under current EU legislation, the use of EIDs is mandatory for all sheep and goat farmers, representing a significant opportunity for the progressive introduction of PFSs into small ruminant production systems, insofar as a mandatory identification infrastructure is already in place that could be leveraged to support individualized feeding protocols [13]. Three main EID application methods have been extensively tested within large-scale European projects, including ear tagging, ruminal bolus, and injectable subcutaneous electronic identification [136,137]. In dairy goat production, behavioral monitoring has traditionally relied on video recording and direct observation to assess lying and standing time and bouts [138]. However, video-based systems present practical limitations in commercial farm settings, including installation complexity, time-intensive video analysis, and potential interference with animal behavior due to observer presence [139]. Automated data loggers offer a promising alternative, providing continuous and detailed behavioral coverage over extended periods [140]. While accelerometers have been successfully applied to monitor lying behavior in cattle, their size has limited their direct application in goats [141,142]. Smaller devices, such as the Hobo Pendant G data logger, have shown potential for goat monitoring, although their validation in this species remains incomplete [143]. As with sheep, these devices should be understood as monitoring technologies that describe behavioral states rather than tools that directly drive feeding decisions; their integration into functional PFS workflows would require coupling with individual identification systems and decision support platforms capable of translating behavioral signals into actionable nutritional responses. Available studies in dairy goats have primarily investigated behavioral responses to social management practices, including separation and reintegration within groups [144], the effects of different stocking densities [145,146], and the impact of environmental enrichment on behavior [147]. Notably, studies specifically examining the use of automated behavioral monitoring as an input to individualized ration adjustment in goats remain scarce, reflecting a broader gap in the translation of monitoring capability into operational precision feeding tools for this species. Further research is therefore needed not only to develop and validate species-specific sensing solutions but more critically to demonstrate integrated systems in which behavioral and identification data jointly inform real-time nutrient delivery decisions under intensive and semi-intensive commercial conditions. Table 5 shows some of the most applied technologies, along with the parameters monitored, in small ruminant.

### 4.2. Monogastric Animals

#### 4.2.1. Swine

Crop resources are facing immense pressure due to competition between animal feed, human food, and bio-industries, which puts great societal pressure on farming [157]. The cost of feed represents a significant portion of production costs for pig farming, with fattening pigs’ feed costs accounting for around two-thirds of total production costs [7] and 15–17% of the production costs for sows and their litter until weaning [158]. To increase the sustainability of pig production, it is crucial to improve the nutrition of pigs. By reducing feed consumption, feed costs can be minimized, and nutrient excretion can be reduced [159]. This, in turn, can have a positive impact on product quality, such as the lean-to-fat ratio, fat quality, and product consistency. Currently, decision making in pig production is based on a combination of animal/environment observations and monthly/quarterly production reports [160]. Precision feeding is a method that can help to better consider individual variability in nutrient requirements within a group. It aims to improve the characterization of individuals or small groups by considering factors such as feed intake, growth potential, body condition, physical activity, and health, among others, to better adapt the quantity, quality, and timing of feed supplied to them [157]. However, the challenge lies in estimating individual requirements and distributing different diets to animals in the same group [161]. Such systems could improve feed and nutrient efficiency, which are significant issues for the sustainability of all pig production systems, whether conventional or alternative.

Individual animal identification is the operational foundation of any precision feeding system in pigs, though the extent to which it has been implemented differs markedly between production categories. Radio-frequency identification (RFID) ear tags allow reliable individual recognition at feeding stations and, when combined with sensors, enable the automation of farm equipment and the transfer of real-time data to farmers or decision support systems capable of making rapid management decisions [162,163].

Electronic feeding stations equipped with RFID readers can record the number of visits, visit time and duration, and the quantity of feed consumed by each individual [164]. In commercial herds, however, individual electronic identification is currently standard practice only for sows, where electronic sow feeding (ESF) stations represent a mature and widely adopted technology [165]. For growing–finishing pigs, individual identification remains uncommon in commercial settings due to the associated costs, even though the technical capability exists and has been demonstrated in research and nucleus selection herds. Body weight monitoring in this category has been approached through various automated technologies, including foreleg weighing systems [166], image analysis [167], machine vision [168,169], and photogrammetry, to determine the three-dimensional shapes estimation [170,171], but these remain predominantly at the research or early-adoption stage rather than representing routine commercial practice. The evidence on the environmental and economic benefits of precision feeding in pigs is promising, though it is important to distinguish between findings obtained in different production contexts. Studies focused on growing–finishing pigs have provided particularly compelling results. Pomar et al. [172] demonstrated that delivering the right amount of feed with the right composition at the right time to each individual pig in the herd can reduce feeding costs by more than 4.6%, while reducing both nitrogen and phosphorus excretion by more than 38% [173], without compromising growth performance. Similarly, Andretta et al. [174] confirmed that feeding growing–finishing pigs with daily tailored diets is an effective approach to reduce nutrient excretion without compromising pig performance or carcass composition, supporting the environmental sustainability of pork production while maintaining productive outcomes [33]. In addition, Pomar et al. [175] found that the concomitant adjustment of dietary nutrient concentrations to match the evaluated requirements of pig populations can significantly reduce feeding costs and nitrogen and phosphorus excretion across the herd. Beyond nutritional efficiency, the integration of precision feeding into large-group production systems offers additional operational advantages, including real-time off-farm monitoring of feed and animals to support optimal slaughter scheduling and production planning, with downstream benefits for animal well-being and meat product quality. It should be noted, however, that the majority of these quantitative estimates derive from experimental or modeling studies; their direct transposition to routine commercial growing–finishing systems—where individual identification infrastructure is largely absent—requires further field-level validation. Table 6 shows some of the most applied technologies in swine, along with the parameters monitored.

#### 4.2.2. Poultry

In poultry production, Abbas et al. [182] stated that smart technology, electronic devices, and operations-controlling gadgets can address most challenges in an organized, efficient, and easy way. With a shortage of labor and land, the use of innovative technology in the poultry industry is indispensable [183]. Today, the competition in production is intense, and it has become necessary to shift from traditional farming systems to modern production [184]. The need for adopting and implementing smart technologies in poultry is evident from the fact that by 2050, a technology-driven poultry farm would be capable of producing and managing 4.1 million data points using sensors and related devices linked through the Internet of Things (IoT) [185].

The implementation of precision feeding systems in poultry production addresses the critical challenge that feed represents approximately 65–70% of the total cost of chicken meat production, making optimization of feeding strategies essential for industry sustainability and profitability [186]. Modern broiler chickens experience rapid growth with daily changes in nutrient requirements, yet conventional production systems typically employ only three to five diet phases, resulting in periods of under- or over-supply of nutrients throughout the production cycle [187]. Precision nutrition addresses this limitation by enabling the blending of dietary components to match the daily requirements of broilers using advanced feeding technologies [11]. Recent studies demonstrate that precision nutrition treatments significantly improve chicken meat production efficiency, with precision feeding systems utilizing only two dietary concentrates blended daily to meet energy and protein requirements, resulting in reduced weight-corrected feed conversion ratios and improved apparent metabolizable energy compared to conventional four-phase feeding programs [8,188]. Precision feeding technologies in broiler production include accurate measurement and distribution of feed quantities adjusted to bird age and breeding purpose, with real-time control of inputs facilitated by electronic load-cell scales and flow meters that monitor feed and water consumption patterns [189,190,191,192]. Advanced systems such as the Kai-Zen robotic feeding system utilize computer algorithms to calibrate feeding rates according to genetics, breed, age, and demand, optimizing feed utilization during production cycles with expected returns on investment of less than one year and potential revenue increases up to 20% [193,194]. These technologies enable real-time dietary adjustments that are particularly effective in broiler systems due to their rapid growth characteristics, while simultaneously reducing nutrient excretion into the environment without compromising growth performance or carcass composition [195,196]. The implementation of precision nutrition regimes targeting daily requirements through on-farm blending of dietary components is anticipated to improve production efficiency, reduce costs, and enhance industry sustainability while providing management benefits including automated feed supply records and reduced ammonia emissions [197]. However, successful implementation requires accurate ingredient characterization, well-defined nutrient databases, and properly established nutrient requirements, representing significant investment challenges that currently limit widespread adoption in commercial operations [195]. Some of the most applied technologies in broiler chicken, along with the parameters monitored, are showed in Table 7.

Advanced precision feeding strategies in laying hens integrate pre-lay and early production nutrition into a unified, performance-based framework. This approach is characterized by reduced dietary energy density, elevated amino acid supplementation, moderate fiber inclusion, and a high proportion of coarse calcium sources. Such formulations have demonstrated improvements in body weight, feed intake, and metabolic adaptation during the critical early laying period [209]. Modern precision nutrition systems enable the continuous monitoring of inter-individual variation in feed efficiency. High feed efficiency hens consume significantly less feed and select diets with greater ash content and lower gross energy compared to their low feed efficiency counterparts, highlighting the value of individual-level nutritional management [210,211]. Complementing dietary strategies, precise environmental control—particularly lighting regimens that regulate circadian rhythms, feeding behavior, and metabolic processes—has been shown to enhance feeding and digestive function, thereby improving feed utilization efficiency compared with conventional commercial lighting schedules. [212]. Similarly, split feeding strategies exploit the physiological mechanisms underlying calcium metabolism: efficient calcium absorption occurs primarily in the duodenum and jejunum, mediated by transporters including TRPV6 and Calbindin-D28K whose expression is regulated by vitamin D_3_. By supplying calcium when it is most physiologically required for eggshell formation, split feeding enables more targeted and efficient nutrient utilization [213].

Despite these advances, the ongoing transition toward cage-free housing systems introduces new management challenges that precision feeding alone cannot fully address. Feather pecking—estimated to affect 40–50% of cage-free flocks—represents a significant welfare and productivity concern [214,215], necessitating the integration of nutritional management with advanced behavioral monitoring technologies. In this context, deep learning models such as YOLOv5 have shown promise for real-time, automated detection and tracking of feather pecking behavior, offering a pathway toward integrated precision systems that couple dietary intervention with continuous welfare surveillance. Table 8 shows some of the most applied technologies in laying hens, along with the parameters monitored.

#### 4.2.3. Fishes

Aquaculture is the fastest-growing food production sector globally, currently supplying over 50% of fish consumed for human nutrition [223]. Intensive fish farming is critically dependent on formulated feeds, which represent 40–70% of total production costs, being a major source of nitrogen and phosphorus load in aquatic environments. Uneaten feed and inefficient nutrient utilization remain primary drivers of water quality deterioration, economic loss, and reduced animal welfare in commercial operations [224]. Precision feeding systems address these challenges by delivering feed in quantities and compositions precisely matched to the real-time appetite and physiological state of fish populations or individual animals, minimizing feed waste while sustaining optimal growth trajectories [225].

Unlike terrestrial livestock, fish exhibit poikilothermic metabolism, meaning their nutritional requirements are profoundly influenced by water temperature, dissolved oxygen concentration, photoperiod, and salinity [226,227]. Feed intake in fish is further modulated by schooling behavior, social hierarchies, and feeding motivation—all of which vary temporally and are difficult to predict using static feeding tables [228]. These biological characteristics define the core design requirements for aquaculture PFSs: feed decision algorithms must integrate real-time environmental and behavioral data to dynamically adjust delivery rate and ration composition.

The concept of satiation-based feeding is central to precision aquaculture nutrition. Fish signal feeding motivation through observable behavioral cues, including increased surface activity and active pursuit of feed particles, while the appearance of uneaten pellets sinking below a defined depth threshold reliably indicates satiation [229]. PFSs exploit these behavioral biomarkers to modulate feed delivery in real time, preventing both underfeeding—which compromises growth—and overfeeding—which generates waste and degrades water quality. Species-specific feeding rhythms must also be incorporated into PFS algorithms: salmonids exhibit distinct diurnal feeding peaks, while warm-water species such as tilapia and sea bass display more continuous appetite patterns [230,231,232,233]. The most widely implemented feed delivery control strategy relies on uneaten pellet detection as a feedback signal. Underwater camera systems capture real-time images of sinking pellets, which are analyzed using computer vision algorithms to quantify waste flux; when pellet flux exceeds a predefined threshold, feed delivery is automatically reduced or halted [234]. Acoustic sensors—specifically echo-sounders and hydro-acoustic transducers—offer a complementary approach by detecting pellet descent via acoustic backscatter, with particular advantage in turbid conditions where optical methods are unreliable [235]. Water quality parameters, including temperature, dissolved oxygen, pH, and ammonia concentration, are continuously monitored and integrated into feed decision algorithms, given that metabolic rate and feed conversion efficiency are directly regulated by these variables [236].

Advanced PFS incorporate stereo-vision systems and deep learning models—particularly convolutional neural networks—for non-invasive estimation of individual fish biomass, enabling continuous recalibration of feeding rations without stressful manual sampling [237]. At the delivery control level, demand feeders—in which fish physically trigger feed dispensing by contacting a pendulum or optical sensor—represent the simplest form of individual-driven precision feeding. More sophisticated dispensers, controlled by programmable logic controllers interfacing with multiparametric sensor arrays, achieve high temporal resolution in feed delivery and are capable of varying pellet size and dietary composition across feeding events [238].

Implementation of PFSs has been consistently associated with reductions in feed conversion ratio (FCR) of 10–30% relative to conventional fixed-schedule feeding across commercially relevant species including Atlantic salmon, rainbow trout, European sea bass, and gilthead sea bream [239,240], translating directly into reduced feed costs and improved economic margins. Correspondingly, precision feeding has demonstrated reductions in feed waste of 15–40% relative to fixed-schedule protocols, with measurable decreases in nitrogen and phosphorus discharge [241,242,243].

It should be noted, however, that PFS architectures developed for pelagic feeders are not universally transferable. Flatfish species exhibit cryptic, bottom-oriented feeding behavior that renders standard pellet-detection algorithms largely inapplicable without significant modification [244,245] while crustacean feeding being nocturnal and spatially diffuse remains poorly amenable to optical monitoring approaches [246]. Addressing these species-specific constraints represents an important frontier for the continued development of feed decision support systems in aquaculture. Table 9 shows some of the most applied technologies in aquaculture, along with the parameters monitored.

### 4.3. Comparative Analysis of Precision Feeding Technologies Across Livestock Species

The technological landscape of precision livestock feeding varies considerably across species, reflecting differences in production systems, individual animal value, and the practical constraints imposed by housing and management conditions [262]. To facilitate cross-species comparison, the degree of technological maturity was assessed using a structured rating framework encompassing four criteria: (i) commercial availability of species-specific PFS solutions; (ii) level of independent scientific validation in peer-reviewed literature; (iii) documented adoption rate at farm level; and (iv) degree of sensor integration and system interoperability. Each criterion was scored on a three-point scale based on the evidence synthesized in the preceding sections, and aggregate scores were used to assign an overall maturity rating as summarized in Table 10. It should be acknowledged that, in the absence of systematic adoption surveys for all species, ratings for less-studied systems—particularly small ruminants and fish—necessarily incorporate a degree of expert judgment and should be interpreted accordingly.

Across this framework, radio-frequency identification (RFID) emerges as the most cross-species technology, being deployed across all six species examined. Its versatility in individual animal identification underpins the functionality of more complex sensor integrations, making it a foundational component in precision feeding architectures regardless of the production system. Beyond RFID, however, the technological profiles diverge substantially.

Dairy cattle achieve the highest maturity rating, supported by a comprehensive suite of commercially available and scientifically validated sensors including accelerometers, load cells, rumen bolus internal sensors, TMR systems, neck collars, and automatic feeders [263]. This breadth of instrumentation reflects the high individual economic value of dairy cows and the intensification of indoor housing systems, which facilitate both sensor deployment and data transmission. Systems based on near-infrared spectroscopy (NIRS), electronic feed intake monitoring, rumination sensors, and IoT-integrated platforms are already widely adopted at farm level [264], though feed composition variability and elevated implementation costs remain non-trivial barriers to universal uptake.

Swine production similarly achieves a high maturity rating, particularly in sow management, where electronic sow feeders (ESFs) represent a well-validated and commercially established solution for individualized feed allocation in group housing [265]. Computer vision and body condition scoring systems further enhance monitoring capacity. Technological development in finisher pigs, however, lags behind, primarily due to the unresolved challenge of reliable individual identification within large, undifferentiated groups [266], which is a limitation that reduces both the validation evidence base and the practical adoption rate for this production stage.

Beef cattle and poultry share an intermediate maturity level, yet for markedly different reasons. In beef systems, the extensive nature of production—particularly in pasture-based contexts—limits the deployment and continuous operation of individual monitoring technologies, constraining both adoption and validation opportunities, even where RFID, accelerometers, and machine vision are technically available [267]. In poultry, the constraint is structural: high stocking density and low per-bird economic value render individual-level monitoring largely impractical. Flock-level approaches mediated by acoustic monitoring, power supply robots (PSRs), feed blending technology (FBT), and machine learning-assisted computer vision represent the current frontier, but independent farm-level validation remains limited [182,205].

The most limited technological readiness is observed in fish and small ruminants, where commercial availability is sparse, peer-reviewed validation is largely confined to experimental or pilot-scale studies, and documented farm-level adoption remains negligible. In aquaculture, physical challenges including underwater sensor degradation due to biofouling, signal transmission limitations in aquatic environments [247], and the exceptional biological diversity of farmed species complicate the generalization of feeding algorithms and hinder the translation of laboratory findings to commercial systems [232,268]. Small ruminant systems, while employing RFID, acoustic monitoring, accelerometers, and GPS-based walk-over weighing, are constrained by the semi-extensive management practices common to sheep and goat farming [129,153] and by the limited availability of commercially validated solutions [140].

Taken together, these comparisons reveal a consistent pattern in which individual animal value, housing intensification, and system controllability are the primary determinants of technological maturity. Species managed under controlled, indoor, high-value production contexts benefit from the most mature and diverse sensor ecosystems, whereas extensive and aquatic systems remain reliant on emerging and largely non-validated technologies. Future research should prioritize the development of low-cost, robust, and species-adapted solutions particularly for aquaculture and small ruminants to bridge the current technological gap and extend the benefits of precision feeding across the full diversity of livestock production systems.

**Table 10:** Synoptic comparison of precision feeding systems across animal species.

Species	Main Technologies/Sensor	Technological Maturity	Main Limitation	References
Dairy cattle	RFID, accelerometer, load cells, weight sensors, Rumen bolus internal sensor, TMR, neck collars, automatic feeders	***** High—commercially available, widely adopted	Feed composition variability; high implementation cost	[73]
Swine	ESF, RFID, load cells, computer vision, body condition scoring systems	**** High—mature in sow management; developing in finishers	Competitive feeding in group housing; individual ID in large groups	[269]
Beef cattle	RFID, accelerometer, machine vision, automatic feeding systems	*** Moderate—growing adoption in intensive feedlots	Extensive management limits individual monitoring; harsh environments	[76]
Poultry	RFID, load cells, acoustic monitoring, accelerometer, PSR, FBT, machine learning computer vision, individual feeder station	*** Moderate—flock-level monitoring well developed	Individual monitoring not feasible; high bird density; low per-bird value	[270]
Fish	RFID, underwater cameras, automatic feeders with pellet detection, computer vision, water quality sensors	** Emerging–low—experimental to early commercial	Underwater sensor constraints; species diversity; biofouling; data transmission	[271]
Small ruminants	RFID, acoustic monitoring, accelerometer, GPS, walk-over weighing	** Emerging-low—mostly experimental	Low individual value; semi-extensive systems; limited commercial solutions	[149]

**: emerging–low level; ***: moderate level; **** and *****: high level of technology maturity. RFID: radio-frequency identification; PSR = power supply robot, FBT = feed blending technology; TMR = total mixed ration; ESF = electronic sow feeders; GPS = Global Positioning System.

## 5. Future Perspective and Conclusions

This review has provided a narrative overview of the current state of sensor-based precision feeding systems across the major animal production species, encompassing both monogastric and polygastric animals. The evidence gathered suggests that PFS technologies hold considerable promise as a transformative approach in modern animal production, enabling a gradual shift from population-level to individual-level nutritional management. It should be acknowledged, however, that the strength of available evidence varies substantially across species: conclusions drawn for dairy cattle and swine are supported by a relatively robust body of commercial-scale validation, whereas inferences regarding beef cattle, poultry, small ruminants, and aquaculture rest predominantly on experimental or pilot-scale studies and should therefore be regarded as preliminary.

Across the species reviewed, PFSs appear capable of improving feed efficiency, reducing production costs, and contributing to environmental sustainability through reduced nutrient waste. The integration of large language models (LLMs) represents one of the most consequential recent developments in this domain, enabling the standardization of heterogeneous data streams from wearable sensors, computer vision systems, automatic feeders, and farm management records into a unified analytical space. Hybrid cloud–edge architectures have emerged in parallel as a practical response to the connectivity constraints of rural environments, with lightweight models embedded in animal-mounted microcontrollers achieving high behavioral recognition accuracy while remote cloud systems handle more computationally intensive optimization tasks. On the biosensor front, the landscape has evolved from simple accelerometers toward passive, multiparameter, minimally invasive devices capable of detecting composite physiological conditions—such as early-stage heat stress—that conventional single-indicator approaches would fail to identify. Digital twins represent the most architecturally ambitious frontier, proposing multi-level coupling from individual animal management to supply-chain traceability; however, no fully operational system has yet been deployed at commercial scale, and existing platforms are more accurately described as prototypes or partial simulation frameworks.

Significant gaps between experimental validation and large-scale commercial deployment nonetheless persist in most sectors beyond dairy and swine, reflecting biological, managerial, and economic constraints that are unlikely to be resolved by technological progress alone. Critical technical challenges—including data standardization, platform interoperability, and high implementation costs—remain to be addressed before the full potential of PFSs can be realized. Adoption is further shaped by farm- and decision-maker-level factors, including farm size, risk perception, and peer influence, which underscore the need for context-sensitive implementation strategies alongside continued technological development.

Beyond technical performance, the widespread adoption of PFSs raises important questions that extend beyond engineering and economic considerations. With respect to data governance, the large volumes of individual-level animal data continuously generated by sensor-based systems present unresolved challenges regarding ownership, privacy, and sharing mechanisms, particularly in contexts where farm-level data may be processed by third-party cloud platforms or integrated into commercial supply-chain traceability systems. Clear regulatory frameworks and transparent data-sharing agreements between farmers, technology providers, and institutional stakeholders will be essential to ensure that data sovereignty remains with the producer and that sensitive operational information is adequately protected. Regarding animal welfare, the automation of feeding decisions introduces risks that merit explicit attention: individual identification errors—whether arising from sensor misreads or algorithmic misclassification—can result in uneven feed allocation, with potential consequences for the nutritional status and health of affected individuals. Furthermore, the deployment of dense sensor networks and continuous monitoring systems may itself constitute a source of behavioral disturbance, particularly in species sensitive to environmental novelty or handling stress. Future PFS development should therefore incorporate validated welfare indicators capable of detecting such system-induced perturbations, and system validation protocols should explicitly account for identification accuracy at the individual level as a welfare-relevant performance metric.

Looking forward, the most plausible trajectory for PFSs is one of progressive integration within broader precision livestock farming ecosystems, in which feeding management is dynamically coordinated with health monitoring, environmental control, and traceability systems. This transition will require standardized data protocols, sustained investment in professional data literacy, and clear regulatory frameworks governing farm-level data ownership. The explicit incorporation of environmental performance indicators—such as nitrogen use efficiency and carbon footprint—as primary PFS outcomes, potentially linked to life cycle assessment frameworks, is identified as a priority research direction. Finally, the development of validated, feeding-specific welfare indicators derived from continuously collected sensor data represents a tractable near-term objective, directly connecting nutritional management decisions to measurable and auditable improvements in animal welfare. Achieving these goals will demand coordinated effort across disciplines, bridging animal nutrition, data science, agricultural engineering, and policy—a convergence that will ultimately determine whether the promise of precision feeding is translated into sustained impact at the farm scale.

## Figures and Tables

**Table 1:** Search strings used with Boolean operators (AND, OR, NOT) used for literature research.

Thematic Clusters	Database	Search Strings
**Precision feeding system in polygastric animals**	Web of Science	“precision feeding” OR “smart feeding” OR “automated feeding” OR “precision nutrition” AND “feed efficiency” OR “nutrient utilization” OR “feed intake” OR “feed conversion” AND “polygastric” OR “ruminant” OR “dairy cow” OR “beef cattle” OR “sheep” OR “goat”
	PubMed	“precision feeding” OR “precision livestock farming” OR “smart feeding” OR “automated feeding” OR “precision nutrition” AND “feed efficiency” OR “nutrient utilization” OR “feed intake” OR “feed conversion” AND “polygastric” OR “ruminant” OR “dairy cow” OR “beef cattle” OR “sheep” OR “goat”
	Scopus	“precision feeding” OR “precision livestock farming” OR “smart feeding” OR “automated feeding” OR “precision nutrition” AND “feed efficiency” OR “nutrient utilization” OR “feed intake” OR “feed conversion” AND “polygastric” OR “ruminant” OR “dairy cow” OR “beef cattle” OR “sheep” OR “goat”
**Precision feeding system in monogastric animals**	Web of Science	“precision feeding” OR “precision livestock farming” OR “smart feeding” OR “automated feeding” OR “precision nutrition” AND “feed efficiency” OR “nutrient utilization” OR “feed intake” OR “feed conversion” AND “monogastric” OR “swine” OR “pig” OR “poultry” OR “broiler” OR “laying hen”
	PubMed	“precision feeding” OR “precision livestock farming” OR “smart feeding” OR “automated feeding” OR “precision nutrition” AND “feed efficiency” OR “nutrient utilization” OR “feed intake” OR “feed conversion” AND “monogastric” OR “swine” OR “pig” OR “poultry” OR “broiler” OR “laying hen”
	Scopus	“precision feeding” OR “precision livestock farming” OR “smart feeding” OR “automated feeding” OR “precision nutrition” AND “feed efficiency” OR “nutrient utilization” OR “feed intake” OR “feed conversion” AND “monogastric” OR “swine” OR “pig” OR “poultry” OR “broiler” OR “laying hen”
**Precision feeding system in aquaculture**	Web of Science	“precision feeding” OR “precision aquaculture” OR “smart feeding” OR “automated feeding” OR “feed dispenser” OR “demand feeder” AND “feed efficiency” OR “feed conversion ratio” OR “feed intake” OR “feeding behavior” OR “feeding behavior” OR “nutrient utilization” AND “aquaculture” OR “fish farming” OR “fish”
	PubMed	“precision feeding” OR “precision aquaculture” OR “smart feeding” OR “automated feeding” OR “feed dispenser” OR “demand feeder” AND “feed efficiency” OR “feed conversion ratio” OR “feed intake” OR “feeding behavior” OR “feeding behavior” OR “nutrient utilization” AND “aquaculture” OR “fish farming” OR “fish”
	Scopus	“precision feeding” OR “precision aquaculture” OR “smart feeding” OR “automated feeding” OR “feed dispenser” OR “demand feeder” AND “feed efficiency” OR “feed conversion ratio” OR “feed intake” OR “feeding behavior” OR “feeding behavior” OR “nutrient utilization” AND “aquaculture” OR “fish farming” OR “fish”
**Technology**	Web of Science	“precision feeding” OR “precision livestock farming” OR “precision aquaculture” OR “smart feeding” OR “automated feeding” AND “artificial intelligence” OR “machine learning” OR “deep learning” OR “computer vision” OR “IoT” OR “sensor” OR “robotic” OR “algorithm” OR “real-time” OR “digital twin” OR “big data” OR “NIR” OR “near-infrared” OR “RFID” OR “wearable”
	PubMed	“precision feeding” OR “precision livestock farming” OR “precision aquaculture” OR “smart feeding” OR “automated feeding” AND “artificial intelligence” OR “machine learning” OR “deep learning” OR “computer vision” OR “IoT” OR “sensor” OR “robotic” OR “algorithm” OR “real-time” OR “digital twin” OR “big data” OR “NIR” OR “near-infrared”
	Scopus	“precision feeding” OR “precision livestock farming” OR “precision aquaculture” OR “smart feeding” OR “automated feeding” AND “artificial intelligence” OR “machine learning” OR “deep learning” OR “computer vision” OR “IoT” OR “sensor” OR “robotic” OR “algorithm” OR “real-time” OR “digital twin” OR “big data” OR “NIR” OR “near-infrared”

**Table 2:** Eligibility criteria: Population, Intervention, Comparison, Outcome, Study (PICOS) design framework.

Element	Included	Excluded
**P, Population**	Cattle, sheep, goats, pigs, poultry, aquaculture	Wild species, duck, turkey, horses, buffalo
**I, Intervention**	Sensor-based PFSs, automated systems, AI/ML applied to power supply	Studies only on dietary formulation without technological component
**C, Comparison**	With/without PFSs; comparison between technologies	**—**
**O, Outcome**	Feed intake, feed efficiency, feeding behavior, benefit, emissions, costs	**—**
**S, Study design**	Research articles, systematic reviews, meta-analysis, book chapters	Abstract, proceedings without peer review, opinions

**Table 3:** Research papers classified by technology used in dairy cattle.

Technologies	Location	Monitored Parameters	Transmission Data System	References
RFID 134 kHz/UHF	Ear tag rumen bolus	Individual identification feeder access	Inductive LF (134.2 kHz)	[72,73]
MEMS: accelerometer triaxial	Neck/leg collar rumen bolus	Motor activity, rumination, estrus detection	BLE/NFC/LoRaWAN	[74,75]
Load cells weight sensors	Feed trough balance	Feed intake, body weight	Wired/Wi-Fi	[76,77]
Rumen bolus internal sensor	Rumen bolus	pH, temperature, rumen movement (SARA)	LoRaWAN/BLE passive RFID	[78,79]
Automatic total mixed ration (TMR)	Feed mixer cattle shed	Distribution of rations with individual program	Wi-Fi/Wired	[80,81]
Neck collars	Neck	Rumination, activity estrus, feeding time	BLE/NFC/Wi-Fi/4G	[82,83]
Automatic feeders	Feed station PMR unit	Concentrate dispensing PMR/individual ration	WiFi/Wired	[84,85]

Abbreviations: BLE = Bluetooth Low Energy; NFC = Near-Field Communication; RFID = radio-frequency identification; TMR = total mixed ration; PMR = partially mixed ration; MEMS = Micro-Electromechanical Systems.

**Table 4:** Research papers classified by technology used in beef cattle.

Technologies	Location	Monitored Parameters	Transmission Data System	References
RFID	Ear tag	Identification, weight	Inductive LF (134 kHz)	[112,113]
Accelerometer MEMS	Ear tag Neck/legs collar	Activity, rumination, estrus cycle	BLE/NFC/4G	[114,115]
Machine Vision/Camera	Feeder/feedlot	Residual feed, feed composition	Wi-Fi/4G cloud	[116,117]
Load cells	Feeder/balance	Individual feed intake, weight	Wired/Wi-Fi	[118,119]
Feeder robot (AFS)	Stable/feedlot	Ration distribution	Wi-Fi	[120,121]
AI/Machine Learning	Cloud/software	Prediction feed intake	Cloud/API	[122,123]

Abbreviations: RFID = radio-frequency identification; MEMS = Micro-Electromechanical Systems; AFS = automatic feeding system; AI = artificial intelligence; BLE = Bluetooth Low Energy.

**Table 5:** Research papers classified by technology used in small ruminants.

Technologies	Location	Monitored Parameters	Transmission Data System	References
RFID	Ear tag; rumen bolus; subcutaneous	Individual identification	Inductive LF (134 kHz)	[148,149]
Acoustic monitoring	Collar	Jaw movements at pasture	BLE/Wi-Fi	[150]
Accelerometer MEMS	Ear tag neck/legs collar	Activity, posture, grazing, rumination	BLE/NFC/LoRaWAN	[151,152]
GPS + Virtual Fencing	Collar	Location, pasture management	GSM/LoRa/4G	[153,154]
WOW	Fixed platform	Body weight	Wired/Wi-Fi	[155,156]

Abbreviations: RFID = radio-frequency identification; MEMS = Micro-Electromechanical Systems; GPS = Global Positioning System; WOW = walk-over weighing; BLE = Bluetooth Low Energy.

**Table 6:** Research papers classified by technology used in swine.

Technologies	Location	Monitored Parameters	Transmission Data System	References
RFID + ESF	Ear tag/stable	Identification, individual feed intake	Inductive HF (13,56 MHz)	[176]
Electronic drinker	Drinking trough	Watering behavior	CAN Bus/Wi-Fi	[177]
Machine Learning/AI	Cloud/edge computing	Forecast requirements, anomalies	Cloud API	[178,179]
CAN Bus + IoT Platform	Infrastructure shed	Multi-feeder control on the network	CAN/Wi-Fi/4G	[180,181]
Automatic feeders	Station/feed silos	Multi-diet ration distribution	Wi-Fi + RFID trigger	[21]

Abbreviations: RFID = radio-frequency identification; ESF = electronic sow feeder; AI = artificial intelligence; CAN = Controller Area Network.

**Table 7:** Research papers classified by technology used in broiler chicken.

Technologies	Location	Monitored Parameters	Transmission Data System	References
Load cells + flow meters	Feeder/water pipe	Feed and water intake (flock)	Wired/Wi-Fi	[192,198]
RFID	Wing band	Identification, weight, feeder access	Inductive HF (13,56 MHz)	[199,200]
Acoustic monitoring	Ceiling microphones	Flock sound, anomalies, estimated weight	Wi-Fi/cloud	[201,202]
Accelerometer MEMS	Wing tag/back	Activity, posture, behavior anomalies	BLE/Wi-Fi	[203,204]
Power supply robot (Kai-Zen)	Warehouse	Calibrated ration distribution	Wi-Fi/algorithm AI	[205,206]
Feed blending technology	Silos/food mixer	Individual ration	Wired/Wi-Fi	[188]
Machine learning/AI	Cloud/edge computing	Growth, FCR, forecast requirements	Cloud API	[207,208]

Abbreviations: RFID = radio-frequency identification; MEMS = Micro-Electromechanical Systems; AI = artificial intelligence; FCR = Feed Conversion Rate; BLE = Bluetooth Low Energy.

**Table 8:** Research papers classified by technology used in laying hens.

Technologies	Location	Monitored Parameters	Transmission Data System	References
RFID	Limb band/back	Identification, feeder access, nest	UHF 860–960 MHz/LF 134 kHz/LoRaWAN	[200,216]
Backpack wearable sensor	Back	Activity, posture, temperature	Wi-Fi/BLE	[203,204]
Load cell + balance	Feeder/smart nest box	Individual body weight, egg weight	Wi-Fi/wired	[217,218]
Computer vision/Kinect 3D	Shed ceiling	Behavior, feeder crowding	Wi-Fi/4G cloud	[219,220]
Feed blending technology	Silos/mixed feed	Individual ration	Wired/Wi-Fi	[221]
Individual feeder station	Individual feeder	Body weight, ration distribution	Wi-Fi + RFID trigger	[222]

Abbreviations: RFID = radio-frequency identification; BLE = Bluetooth Low Energy; LoRaWAN = Long-Range Wide Area Network.

**Table 9:** Research papers classified by technology used in aquaculture.

Technologies	Location	Monitored Parameters	Transmission Data System	References
Computer vision	Underwater and surface cameras	Feeding behavior, growth, residual feed	Wi-Fi/4G/edge computing	[247,248]
Acoustic monitoring/hydrophone	Tanks/cages	Food intensity, behavior, biomass	Wired/Wi-Fi	[249,250]
Water quality sensors IoT	Underwater sensor	pH, dissolved O_2_, T°, salinity, NH_3_, CO_2_	Wi-Fi/ZigBee/LoRaWAN	[236,251]
Implantable biosensors	Implanted in fish	Heart rate, visceral T°, acceleration	Wireless underwater	[252,253]
Acoustic tag system (ATS)	Tag on fish	3D position, trajectory, behavior	Hydrophones/passive acoustic	[254,255]
AFS	Tanks/cages	Calibrated ration disbursement	Wi-Fi/algorithm AI	[256,257]
RAS + underwater cameras	Tanks	Images every 5 s, density, average weight	Wi-Fi/wired	[258,259]
AUV/ROV underwater	cages/tanks	Biomass inspection, composition-testing	Underwater acoustic/4G	[260,261]

Abbreviations: IoT = Internet of Things; AFS = automatic feeding system; RAS = Recirculating Aquaculture Systems; AUV = Autonomous Underwater Vehicle; ROV = Remotely Operated Vehicle; T = temperature.

## Data Availability

No new data were created or analyzed in this study. Data sharing is not applicable to this article.

## Abbreviations

The following abbreviations are used in this manuscript:

ADG	Average Daily Gain
AFS	Automatic Feeding System
AI	Artificial Intelligence
APIs	Application Programming Interfaces
AMS	Automatic Milking Systems
AUV	Autonomous Underwater Vehicle
BLE	Bluetooth Low Energy
CAN	Controller Area Network
CNNs	Convolutional Neural Networks
DM	Dry Matter
DMI	Dry Matter Intake
DSS	Decision Support System
EID	Electronic Identification
ESF	Electronic Sow Feeding
FBT	Feed Blending Technology
FCR	Feed Conversion Rate
FMIS	Farm Management Information Systems
GHG	Greenhouse Gas
GPS	Global Positioning System
ICT	Information and Communication Technologies
IoT	Internet of Things
LCA	Life Cycle Assessment
LLMs	Large Language Models
LoRaWAN	Long-Range Wide Area Network
MEMS	Micro-Electromechanical Systems
ML	Machine Learning
NFC	Near-Field Communication
NIR	Near-infrared
NIRS	Near-infrared Spectroscopy
NPS	Noseband Pressure Sensor
PDO	Protected Designation of Origin
PDT	Precision Dairy Technology
PFS	Precision Feeding Systems
PICOS	Population, Intervention, Comparison, Outcome, Study
PLCs	Programmable Logic Controllers
PLF	Precision Livestock Farming
PMR	Partially Mixed Ration
PSR	Power Supply Robot
RAS	Recirculating Aquaculture Systems
RFI	Residual Feed Intake
RFID	Radio-Frequency Identification
RIC	Roughage Intake Control
ROV	Remotely Operated Vehicle
SGR	Specific Growth Rate
TGC	Thermal Growth Coefficient
TMR	Total Mixed Ration
WOW	Walk-Over Weighing
